# Biomimetic Nacre-like Hydroxyapatite/Polymer Composites for Bone Implants

**DOI:** 10.3390/jfb14080393

**Published:** 2023-07-25

**Authors:** Parinaz Tabrizian, Huijun Sun, Urangua Jargalsaikhan, Tan Sui, Sean Davis, Bo Su

**Affiliations:** 1 Biomaterials Engineering Group, Bristol Dental School, University of Bristol, Bristol BS1 2LY, UK; yg20581@bristol.ac.uk (P.T.);; 2 School of Mechanical Engineering Sciences, University of Surrey, Guildford GU2 7XH, UK; 3 School of Chemistry, University of Bristol, Bristol BS8 1TS, UK

**Keywords:** biomimetic, bioactive, hydroxyapatite, nacre, bi-directional freeze-casting, nacre-like composite, bone implants, mechanical properties, fracture toughness

## Abstract

One of the most ambitious goals for bone implants is to improve bioactivity, incapability, and mechanical properties; to reduce the need for further surgery; and increase efficiency. Hydroxyapatite (HA), the main inorganic component of bones and teeth, has high biocompatibility but is weak and brittle material. Cortical bone is composed of 70% calcium phosphate (CaP) and 30% collagen and forms a complex hierarchical structure with anisotropic and lamellar microstructure (osteons) which makes bone a light, strong, tough, and durable material that can support large loads. However, imitation of concentric lamellar structure of osteons is difficult to achieve in fabrication. Nacre from mollusk shells with layered structures has now become the archetype of the natural “model” for bio-inspired materials. Incorporating a nacre-like layered structure into bone implants can enhance their mechanical strength, toughness, and durability, reducing the risk of implant catastrophic failure or fracture. The layered structure of nacre-like HA/polymer composites possess high strength, toughness, and tunable stiffness which matches that of bone. The nacre-like HA/polymer composites should also possess excellent biocompatibility and bioactivity which facilitate the bonding of the implant with the surrounding bone, leading to improved implant stability and long-term success. To achieve this, a bi-directional freeze-casting technique was used to produce elongated lamellar HA were further densified and infiltrated with polymer to produce nacre-like HA/polymer composites with high strength and fracture toughness. Mechanical characterization shows that increasing the ceramic fractions in the composite increases the density of the mineral bridges, resulting in higher flexural and compressive strength. The nacre-like HA/(methyl methacrylate (MMA) + 5 wt.% acrylic acid (AA)) composites with a ceramic fraction of 80 vol.% showed a flexural strength of 158 ± 7.02 MPa and a Young’s modulus of 24 ± 4.34 GPa, compared with 130 ± 5.82 MPa and 19.75 ± 2.38 GPa, in the composite of HA/PMMA, due to the higher strength of the polymer and the interface of the composite. The fracture toughness in the composition of 5 wt.% PAA to PMMA improves from 3.023 ± 0.98 MPa·m^1/2^ to 5.27 ± 1.033 MPa·m^1/2^ by increasing the ceramic fraction from 70 vol.% to 80 vol.%, respectively.

## 1. Introduction

After decades of research on bioactive implants for bone repair and regeneration, replicating the mechanical strength and toughness of cortical bone is still a challenge for engineers and clinicians [1,2,3]. A perfect candidate biomaterial has biocompatibility and mechanical properties similar to the bone in which it is implanted, such as Young’s modulus, high tensile strength, stiffness, and fatigue resistance [4,5]. The most commonly used materials for bone implants such as rods, screws, spinal fusion cages, and plates are stainless steel (SS), titanium (Ti), and polyetheretherketone (PEEK). However, each of these materials also has its drawbacks, such as problems with stress-shielding in Ti and SS implants and poor osteoconductivity in PEEK [3,6]. Therefore, there is a tremendous need to find bioactive and biocompatible materials with bone-like stiffness, strength, and toughness, that can prevent further postoperative complications, like limitation of range of motion, reduce pain, and minimize the need for additional surgery [1,6]. 

Bone tissue can be divided into two types: cortical (or dense bone) and cancellous (or spongy bone). Cortical bone has a porosity of 5–15%, while the porosity of cancellous bone ranges from 40–95%. Cortical bone is composed of 70% calcium phosphate (CaP) and 30% collagen, which forms a complex hierarchical structure with anisotropic and lamellar microstructures. Osteons have a lamellar structure with a thickness of 3–7 μm surrounded by blood vessels and nerves. Osteons are highly mineralized concentric bone layers composed of aligned collagen fibrils, which can be considered as reinforcing and toughening microelements. This structure makes bone a light, strong, tough, and flexible material that can support large loads. The toughness of bone is the result of mutual competition between extrinsic (crack-tip shielding) toughening mechanisms and intrinsic (plastic deformation) toughening mechanisms. Intrinsic toughening mechanisms, such as sliding of collagen fibrils and nucleation of micro- and nano-scale damages, are defined as those that confer resistance to microstructural perturbations upstream of the crack tip. Extrinsic toughening mechanisms, such as crack bridging and crack deflection reduce the driving force contributing to crack propagation. The preferential orientation of osteons in cortical bone provides an effective extrinsic toughness that is anisotropic. A crack that propagates perpendicular to the osteons is more likely to be deflected and twisted than a crack that propagate parallel to the osteons and thus may explain the anisotropy of fracture toughness [1,3,6]. 

To develop synthetic biomaterial composites that can be used as bone implants, it is necessary to imitate bone-like structures to reach the mechanical properties and other biological functionalities of real bone. Biological bone tissues indeed tend to have whisker-like structures at nanoscales which consist of mineralized collagen nanofibers. The preparation of such bone-like HA as a scaffold is essential to enhance the bone repair biologically. In addition to bioactivity and biocompatibility, mimicking the hierarchical structure of natural bone provides load-bearing capacity in the implant and minimizes stress concentration. So, the composite exhibits similar mechanical properties to natural bone with long-term stability, enhancing the lifespan of the implant and functionality for the patient [7,8,9].

In this regard, the comparison between bone and nacre structure shows good similarity in terms of hierarchical microstructure and the toughening mechanisms through which they operate [8].

So, nacre-like ceramic composites have opened new horizons for the fabrication of biomimetic bone implants with high strength and toughness [5,6]. In the nacre structure of seashells, a mechanism of toughness occurs based on the creation of weak interfaces where cracks can be deflected and energy is dissipated by opening surfaces, resulting in higher fracture toughness in ceramic constituents [10]. So, a good example of tough composites are nacre-like composites, that mimic the brick-and-mortar microstructure of seashells. Based on the design of the microstructure, the composite exhibits a rising resistance curve (R-curve) indicating a higher amount of energy is required to propagate a crack. Various methods have been used to develop nacre-like composites with hierarchical brick-and-mortar microstructure, of which bi-directional freeze-casting has proven to be a powerful method for producing bulk composites with excellent mechanical properties [11,12]. The bi-directional freeze-casting method is a modification of the unidirectional freeze-casting method, a polydimethylsiloxane (PDMS) wedge with different slopes is placed between the suspension and the cold finger [10,13]. Due to the low thermal conductivity of the PDMS wedge, the thinner side cools faster during freezing, resulting in a temperature gradient not only in the vertical but also in the horizontal direction. The ice crystals nucleate only at the bottom of the wedge and continue to grow preferentially in two directions. Bi-directional freeze-casting is an effective method for generating long-range aligned lamellar structures, and has already been used to build various functional building blocks into nacre-like composites [11,13,14]. 

In terms of materials, hydroxyapatite (HA) is the most stable calcium phosphate phase with a Ca/P ratio of 1.67. It is formed in nature and can be extracted, but various ions and vacancies form defective structures. Its solubility is low in physiological environments determined by temperature, pH, body fluids, etc. Hydroxyapatite is considered a bioactive material and is a mineral form of calcium apatite that closely resembles the composition and structure of natural bone minerals. It is the primary inorganic component of human bones and teeth. When used in medical and dental applications, hydroxyapatite demonstrates bioactive properties, meaning it can interact with living tissues and promote biological activity. This bioactivity is mainly due to the chemical similarity between hydroxyapatite and the mineral component of bones and teeth. In terms of contacting living tissue, such as bone or tooth surfaces, it can bond to the surrounding tissues through osseointegration. This integration enables the formation of a direct chemical and mechanical bond between the hydroxyapatite material and the surrounding bone, facilitating bone growth and regeneration [15,16].

Hydroxyapatite is commonly used in various biomedical applications, including bone grafts, dental implants, and coatings for orthopedic implants. Its bioactive nature makes it a desirable material for promoting tissue regeneration and improving the long-term success and stability of implantable medical devices [17,18,19].

The surface of HA can serve as a nucleus for bone minerals in body fluid, which do not cause inflammatory reactions when used clinically. They are osteoconductive but not osteoinductive. However, they are mechanically weak and brittle and exhibit weaknesses in strength and toughness, making it difficult to achieve the mechanical properties of cortical bone [16,20]. 

Methyl methacrylate (MMA) is an important monomer, which is widely used for producing polymethyl methacrylate in different biomedical applications, such bone cements, screw fixation in bone, and filler for bone cavities and skull defects [21,22]. To fabricate nacre-like composite, PMMA with low viscosity and high strength makes it an appropriate polymer phase compared to synthetic polymer like PLA, which can infiltrate the ceramic scaffold to make the final brick-and-mortar microstructure. Based on the literature, by infiltrating freeze-cast alumina scaffolds with poly (methyl methacrylate) (PMMA) as a compliant layer, bending strengths of 210 MPa with remarkable fracture toughness have been achieved that exceed a stress intensity of 30 MPa [23]. PMMA can replace ceramic materials in areas where higher strength and toughness are preferred. Resulting in a nacre-like SiC/PMMA composite, it reveals a rising crack resistance (R-curve) behavior where the toughness increases with crack extension [24]. Investigations into the role of the polymer phase in the mechanics of nacre-like composites show that the polymer phase has a resounding impact on the mechanical performance of nacre-like composites. The composite strength significantly increases with stiffer polymers like polyether urethane diacrylate-co-poly(2-hydroxyethyl methacrylate) (PUA-PHEMA) and poly(methyl methacrylate) (PMMA) by avoiding the stress concentrations at the mineral bridges [25]. Research shows that adding acrylic acid (AA) to MMA can modify the properties of PMMA and may also imply the onset of new characteristics. Thus, the copolymerization of MMA with acrylic acid (AA) has been intensively studied, and a variety of materials with useful performance features have been obtained with potential medical applications for regeneration of soft tissues [21,26]. A copolymer of polymethyl methacrylate (PMMA) and polyacrylic acid (PAA) can exhibit varying degrees of biocompatibility and biodegradability. PMMA is generally considered to be biocompatible and has been used in various medical applications. PAA, on the other hand, is also generally biocompatible. When PMMA and PAA are combined in a copolymer, resulting in a biocompatible material. The incorporation of PAA into a copolymer can introduce biodegradability to some extent. PAA is known to be biodegradable under certain conditions, and by combining with PMMA, it can improve the biological activity of the final composite as well [27,28].

In this study, the authors investigate and fabricate HA/polymer composites to improve the brittleness and weaknesses of bioactive HA materials by mimicking the brick-and-mortar structure of nacre through a bi-directional freeze-casting method. The effective processing parameters for the mechanical properties of the composites were studied, and the effects of the ceramic, interface, and polymer phases were cited as the most important parameters for producing bone-like strength and tough, nacre-like HA/polymer composites that could potentially be used in orthopedics such as spinal fusion and bone fracture fixation implants.

## 2. Materials and Methods

### 2.1. Slurry Preparation

Hydroxyapatite (Captal S, Plasma biotal, Buxton, UK) with an average particle size of 2.5 µm was used as the ceramic material. Darvan 821A (R. T. Vanderbilt Co., Norwalk, CT, USA) served as a dispersant that aided solute colloid formation, and polyvinyl alcohol (PVA powder, MW: 30,000–70,000, Sigma Aldrich, Dorset, UK) was used as a binder. The slurry contained 15 vol.% HA with 1.5 wt.% dispersant and 6 wt.% binder based on solids loading were dispersed in deionized water and transferred to a polyethylene (PE) bottle containing zirconia grinding balls and then milled (1600-VS-A, Pascal engineering, Crawley, UK) for 24–48 h at ambient temperature and high speed (~200 rpm). The amount of solid loading, dispersant, and binder were optimized based on the rheological behavior and processability of the sample after freeze-drying. After ball milling, the slurries were degassed by adding 0.1 mL octanol for 30 min to remove the air bubbles generated during slow ball-milling.

### 2.2. Fabrication of Nacre-Like HA/Polymer Composites

The slurry was poured into the PDMS molds with a slope angle of 10° and covered with silicone to obtain a flat surface at the bottom of the sample. Freezing started at the top line of the copper wedge and ended at the other side of the mold. The technical details of bi-directional freeze-casting were reported in a previous work [29]. After freezing, the ice formed in the frozen samples was sublimated in a freeze dryer (Lyotrap, Lte Scientific Ltd., Oldham, UK) at −60 °C for at least 48 h for each sample under a vacuum pressure of 0.03 mbar. To control the ceramic fraction in the final composites, highly aligned lamellar ceramic scaffolds were densified by a hydraulic press (PerkinElmer, Waltham, MA, USA). Finally, a furnace with an oxidation atmosphere was used to sinter the green bodies; in the first stage, the binder was burned out at 600 °C for 2 h, followed by dwelling at 1300 °C for 4 h. The sintering procedure was determined based on the literature [5,30,31]. The as-prepared scaffold with its long-range aligned lamellar structure was first grafted with γ–methacryloxypropyltrimethoxysilane (γ–MPS, Sigma Aldrich, Dorset, UK) in ethanol at different concentrations and for different time periods. Characterization of the grafted ceramic surface was performed by using an attenuated total reflectance (ATR) FTIR spectrometer (PerkinElmer, spectrum-one FTIR, Waltham, MA, USA). In addition, the hydrophobicity of the grafted surface was measured using a drop shape analyzer (DSA100, KRUSS, Hamburg, Germany). Subsequently, the grafted scaffolds were infiltrated with a solution of methyl methacrylate (MMA, Sigma Aldrich, Dorset, UK) and 0.5–1 wt.% azobisisobutyronitrile (AIBN, Sigma Aldrich, UK) as an initiator, and acrylic acid (AA, Sigma Aldrich, Dorset, UK) as a second polymer phase in Cast’ N Vac for 2 h, then heated at 45 °C for 24 h to complete polymerization, and annealed at 90 °C for 2 h.

### 2.3. Characterization

A Zwick Roell universal testing machine (Z020, Zwick Roell, Ulm, Germany) was used to determine compressive and flexural strength (ASTM Standard C1424-15 and D790-15) [32]. Five specimens were tested for each condition. Porosity was measured by the Archimedes method according to the standard (ASTM B962-17) [33]. Scanning electron microscope (EM, Quanta 400-FEI Scanning Electron Microscope, San Diego, CA, USA) was used to study the morphology of the different specimens. X-ray diffraction (XRD, Bruker D8 Advance, Coventry, UK), with Cu-Ka (λ = 1.54 Å) was conducted to investigate any potential changes in the crystalline phase. The fracture toughness of the specimens was measured by three-point bending on single-edged notched bending (SENB) with ASTM E1820 [34]. The prepared SENB specimens were tested in situ at SEM using a Deben Micro-Test 150 N bending stage (Deben, Bury Saint Edmunds, UK) with a support span of 20 mm and a displacement rate for loading and/unloading of 0.1 mm/min. In situ SEM was used to monitor crack propagation in the real-time and record high resolution images after each loading and/unloading cycle.

Based on standard fracture mechanics, the post-fracture dissipated energy (J-integral) was calculated as a function of crack extension with an overall straight trajectory of the crack over the specimen depth (mode I). Fracture toughness, K_J_, was determined by back-calculation from the equivalence of mode I, J–K:

(1) $$K_{J}=\sqrt{J\;E^{\prime }}$$

The J-integral was formed from the elastic and plastic components as follows:

(2) $$J=J_{\mathrm{el}}+J_{\mathrm{pl}\;}$$

The elastic contribution was calculated using the theory of linear-elastic fracture mechanics:

(3) $$J_{\mathrm{el}}=K^{2}/\;EE^{\prime }$$

where E′ is the elastic modulus of the composite under pure strain conditions and is expressed as follows:

(4) $$E^{\prime }=E/{\left(1-\nu \right)}^{2\;}$$

Here, E is the modulus of elasticity of the composite, and ν is the Poisson’s ratio. E was determined using the rule of mixtures, and ν = 0.3 was used for all composites. The mode I stress-intensity K was calculated as:

(5) $$K=\frac{\mathrm{PS}}{{\mathrm{BW}}^{\frac{3}{2}}}f\left(\frac{a}{w}\right)$$

(6) $$f\left(\frac{a}{w}\right)=\frac{3{\left(\frac{a}{w}\right)}^{\frac{1}{2}}\left[1.99-\left(\frac{a}{w}\right)\left(1-\frac{a}{w}\right)\left(2.15-3.93\left(\frac{a}{w}\right)+2.7{\left(\frac{a}{w}\right)}^{2}\right)\right]}{2\left(1+2\frac{a}{w}\right){\left(1-\frac{a}{w}\right)}^{\frac{3}{2}}}$$

where P is the maximum load (N), S is the span (mm), B is the specimen thickness (mm), W is the specimen width (mm), and a is the crack length (mm).

The plastic component $J_{\mathrm{pl}}$ was defined as:

(7) $$J_{\mathrm{pl}}=1.9A_{\mathrm{pl}}/\mathrm{Bb}$$

where b is the uncracked ligament, and A_pl_ is the plastic area under the curve between loading and plastic displacement. According to ASTM, the maximum crack extension for a specimen is given by $∆a_{\max}=0.25b_{0}$, where b_0_ is the initial uncracked ligament determined as $b_{0}=\;W-{\;a}_{0}$. Only the crack expansions within the range valid according to ASTM are included in the linear fit [34,35,36]. 

## 3. Results

### 3.1. Effect of Ceramic Phase

**Figure 1:**
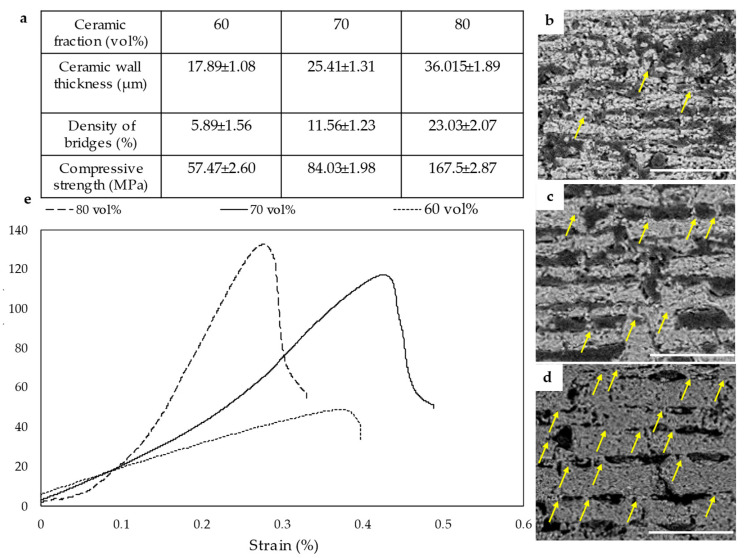
(**a**) Effect of ceramic fraction on ceramic wall thicknesses, ceramic bridges density, and compressive strength. Increasing the ceramic fraction from 60 vol.% to 80 vol.%, results in an increase in wall thickness to 36.01 ± 1.89 µm with a higher ceramic bridges density of 23.03 ± 2.07 (%) and a compressive strength of 167.5 ± 2.87 MPa. (**b**–**d**) show the microstructure of the composite at ceramic fractions of 60 vol.%, 70 vol.%, and 80 vol.%, respectively (Scale bars are 500 µm). (**d**) The yellow arrows show the ceramic bridges increase by increasing the ceramic fraction. (**e**) Stress–strain diagram of different composites with different ceramic fractions. Flexural strength increases to 130 ± 5.82 MPa and the Young’s modulus is 19.75 ± 2.38 GPa for composites with 80 vol.%.

After sublimation and sintering, an HA scaffold with a long-range lamellar structure with 70% porosity was obtained. The scaffold was further densified by uniaxial pressing to ≈20–40% porosity. Figure 1a illustrates the effects of ceramic fraction on wall thickness, density of ceramic bridges, and compressive strength. The average value of five specimens for each ceramic fraction was given. Increasing the ceramic fraction from 60 vol.% to 80 vol.% increases the wall thickness from 17.89 ± 1.08 to 36.01 ± 1.89 µm with higher bridges density and compressive strength of 167.5 ± 2.87, which was 57.47 ± 2.60 MPa. Figure 1b–d show the microstructure of the composites based on ceramic fractions of 60 vol.%, 70 vol.%, and 80 vol.%, respectively. By increasing the ceramic fractions, the wall becomes longer and thicker; moreover, the density of ceramic bridges increases from 5.89 ± 1.56 to 23.03 ± 2.07 (%), which is crucial for improving the strength and toughness of the final composite because they can transfer and redistribute stresses and enhance the frictional sliding between the ceramic layers, leading to improved mechanical properties. (d. The yellow arrows show the increase in ceramic bridges due to the increase in the ceramic fractions) [37,38]. The ceramic bridges are formed due to the conflict between forced and preferential ice growth during bi-directional freeze-casting, resulting in an oblique ice growth direction [39,40].

Increasing the ceramic fraction to more than 80 vol.% destroyed the brick-and-mortar as the ceramic walls fused together and the distinct layered structure was lost in the subsequent sintering stage. Figure 1e shows the stress–strain curve of HA/PMMA composites at different ceramic fractions. The composites with different ceramic fractions showed similar failure mechanisms but different strength values, the composite with 80 vol.% having a flexural strength of 130 ± 5.82 MPa and Young’s modulus of 19.75 ± 2.38 GPa. However, the composite with 70 vol.% ceramic fractions had a flexural strength of 115 ± 2.67 MPa and Young’s modulus of 14.36 ± 2.38 GPa. These values were 52.68 ± 3.78 MPa and 10.11 ± 1.23 GPa for 60 vol.% ceramic fractions. Thus, higher ceramic fractions increase the density of the ceramic walls and the mineral bridges, resulting in a stronger composite.

### 3.2. Effect of Interface

To produce a nacre-like ceramic composite, it is important to improve the interface between the ceramic and the polymer. To this end, the silanization process is an important procedure to establish a strong interface between the ceramic and polymer phases. HA itself has O–H groups in the surface chemistry, which can establish a bond with the silane group. Therefore, it is important to optimize the grafting of the HA scaffold to find the best bonding and flexural strength based on Fourier-transform infrared spectroscopy (FTIR) and contact angle measurement, which can confirm the silanization of the ceramic surface.

**Figure 2:**
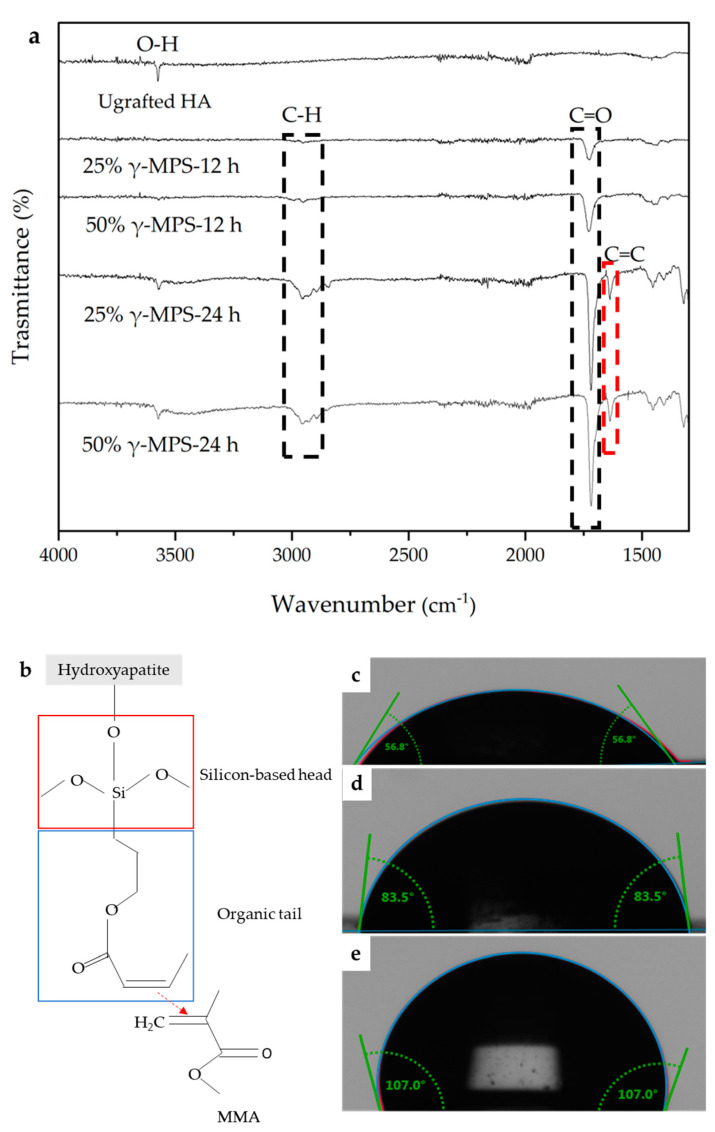
(**a**) FTIR spectra of silane-grafted hydroxyapatite with ɣ–MPS in ethanol at different concentrations and soaking times. (**b**) The schematic of silanization process and effect of silanization conditions. The C=O and C=C found in FTIR spectra show the organic tail on hydroxyapatite surface after grafting with ɣ–MPS. The contact angle images of the different samples show, (**c**) non-grafted HA scaffold, (**d**) ethanol with 25 wt.% ɣ–MPS after 24 h, and (**e**) ethanol with 50 wt.% ɣ–MPS after 24 h. It can be seen that increasing the ratio of ɣ–MPS improves the hydrophobicity of the scaffold surface, which leads to stronger adhesion of the ceramic and polymer phases and thus better mechanical properties.

Figure 2a shows the results of Fourier-transform infrared spectroscopy (FTIR) in the region of 4000–1000 cm^−1^ and contact angle analysis after silanization in different ratios of 25 wt.% and 50 wt.% ɣ–MPS/ethanol solutions at both grafting times of 12 h and 24 h. The silane coupling agent usually acts as a kind of mediator that connects organic materials with inorganic materials. The silane molecule consists of a silicon-based head and an organic tail that is linked to inorganic and organic phases, respectively. In particular, the silicon-based head can firmly bond to reactive groups on a substrate surface (e.g., glass, metal, and ceramic) via a covalent bond. In other words, the O–H (hydroxyl group) on the HA surface reacts with the –Si–OR (R is –CH_3_ for ɣ–MPS) on the head of the silane molecule and then generates H–OR and the silane-grafted HA scaffold. 

As shown in all ratios and grafting times compared to the non-grafted scaffold, there is no peak and no change on non-grafted scaffold. The peaks at 1630 cm^−1^ were assigned to the stretching bonds of carbon–oxygen double bonds (C=O), which were seen in all spectra at all concentrations or times, and at 1720 cm^−1^, the carbon–carbon double bonds (C=C) were assigned with increasing the time from 12 h to 24 h. Based on FTIR analysis, this strong bond can occur in all configurations, but it was developed with time, which can possibly show reaction progress through the conversion of a different functional group into a double bond. The C–H stretching bonds was localized at 2980 cm^−1^ and relates to the –CH_2_CH_2_– on the silane molecule determined after 24 h grafting for both concentrations of 25 wt.% and 50 wt.% ɣ-MPS in ethanol. Figure 2b shows the schematic of silanization process and effect of silanization conditions. The C=O and C=C found in FTIR spectra show the organic tail on hydroxyapatite surface after grafting with ɣ-MPS. Based on FTIR results, the silanization of the HA scaffold depends on the time and concentration of ɣ–MPS. Longer immersion time and higher concentration of ɣ–MPS results in more complete coverage of hydrophobic organic tails, leading to more covalent bond formation and strong interface. 

The analysis of the contact angle of the grafted scaffold can confirm the hydrophobicity of the surface which can lead to the optimization of the degree of silanization to achieve a stronger bond between the ceramic and the polymer. Figure 2c–e show the effect of silanization conditions on the hydrophobicity behavior of the scaffold. As shown in Figure 2d the contact angles increase to the 83.5° due to silanization at the concentration of 25 wt.% ɣ–MPS grafted for 24 h; however, when the concentration of ɣ–MPS is increased to 50 wt.%, the angle reaches 107°, which shows hydrophobicity of the surface, which is due to the grafting with a higher concentration of ɣ–MPS with functional carbon double bonds. Thus, the ceramic surface forms covalent bonds with the polymer phase, resulting in stronger interface between the polymer and ceramic phases and improving the strength of the final composite. 

**Figure 3:**
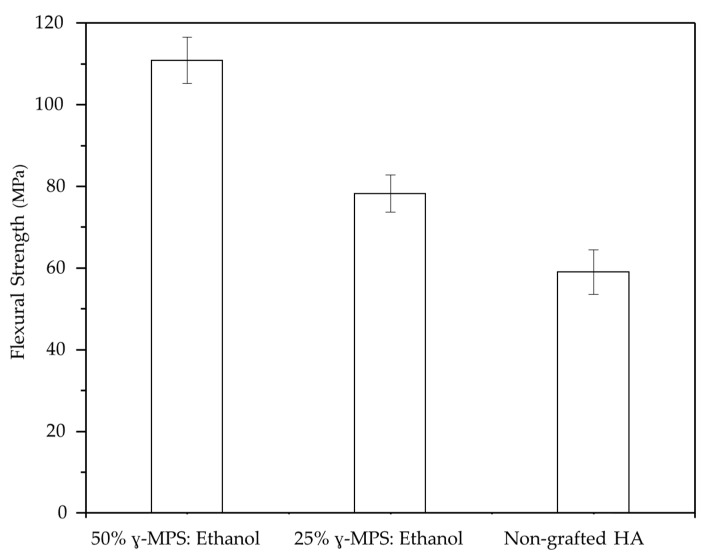
Flexural strength is based on different situations of silanization process. The maximum value of the strength reaches 117 ± 1.89 MPa for the sample silanized with the highest concentration of salinization solution and soaked for 24 h.

Figure 3 shows how silanization affects the flexural strength of the final composite at a constant ceramic fraction of 70 vol.%. Based on the FTIR and contact angle analysis, the maximum flexural strength of 117 ± 1.89 MPa belongs to the composite where the scaffold was silanized in 50 wt.% ɣ–MPS for 24 h which shows stretching bonds of carbon–carbon double bonds (C=C), carbon–oxygen double bonds (C=O), and carbon–hydrogen stretching bonds (C–H) with a hydrophobic surface, resulting in stronger interface bonds between the ceramic and the polymer.

### 3.3. Effect of Polymer Phase 

**Figure 4:**
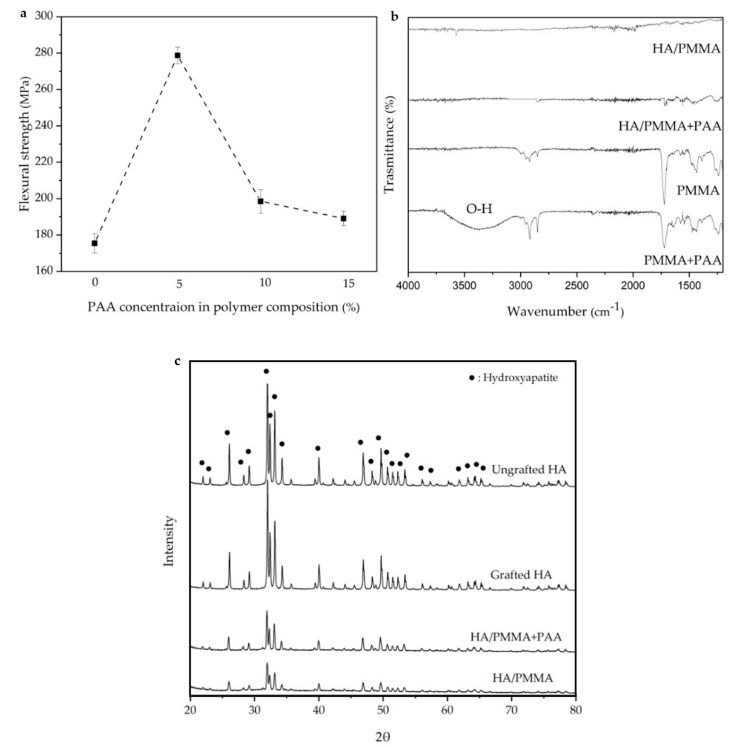
(**a**) The effect of adding different amounts of PAA to PMMA on the flexural strength of the polymer phase. (**b**) The FTIR results of the HA/PMMA, the different polymer phases, and HA/PMMA + PAA. (**c**) The XRD spectra of the ungrafted HA, grafted HA, HA/PMMA + PAA, HA/PMMA, show that hydroxyapatite is the main crystalline phase and silanization and polymerization do not have effect on peak shifting or phase changing and the crystallinity is identical.

**Figure 5:**
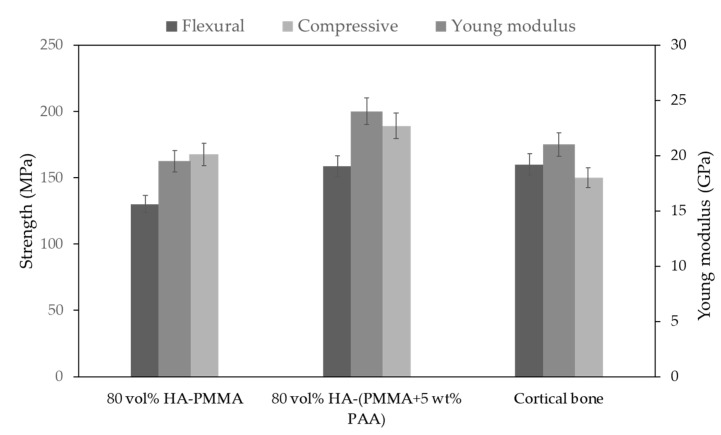
The effect of the addition of PAA on the mechanical properties of HA/PMMA with the same ceramic fraction composites. The flexural, compressive strength, and Young’s modulus increase to 158 ± 7.02, 189.09 ± 6.45 MPa, and 24 ± 4.34 GPa, respectively, compared with the value of cortical bone of 160, 150 MPa, and 21 GPa, respectively. The mechanical data of the cortical bone were taken from the literature [41,42,43].

The polymer phase has a resounding influence on the mechanical properties of a nacre-like composite. Figure 4a shows the effects of adding PAA to PMMA on the flexural strength of the pure polymer. As shown by the addition of 5, 10, and 15 wt.% acrylic acid to the polymer composition, the flexural strength increases compared to pure PMMA; however, the results show that the optimum value in terms of highest flexural strength is 5 wt.% PAA. The presence of AA can form a covalent bond with the free radical of MMA, which increases flexibility and allow stronger chain and elongation. Figure 4b shows the FTIR results of HA/PMMA, the different polymer systems and the HA/PMMA + PAA. Based on the FTIR, the combination of PMMA and PAA shows the presence of hydroxyl group (O–H) around 3500 cm^−1^. In the composite spectra, there is no hydroxyl peak (O–H), which shows that O-H groups introduce new bonds in the interface of ceramic and polymer, resulting in stronger interfacial connection. Figure 4c shows the X-ray diffraction (XRD) spectra of the ungrafted HA, grafted HA, HA/PMMA + PAA, and HA/PMMA exhibit consistent patterns, indicating that hydroxyapatite remains the predominant crystalline phase. The results from the XRD analyses indicate that neither the silanization process nor the subsequent polymerization processes exert any discernible influence on peak shifting or phase transformation, so the crystallinity of the samples remains un-changed throughout the silanization and polymerization procedures. Therefore, the addition of PAA to the polymer phase not only makes strong copolymer, but also with introducing new bonds in the interface improves interfacial strength, which can increase the mechanical properties of final composite shown in Figure 5. 

From Figure 4a it can be seen that the addition of 5 wt.% PAA to PMMA is an optimum level for the use of PAA in the polymer phase. Too high addition of PAA would lead to an increase in stresses at the interfaces, resulting in lower flexural strength. At 5 wt.% PAA, the mechanical properties improved, for instance the flexural strength increases from 130 ± 5.82 to 158 ± 7.02 MPa shown in Figure 5, compared to the cortical bone values of 160 MPa which can be considered a good candidate for future bone implants.

**Figure 6:**
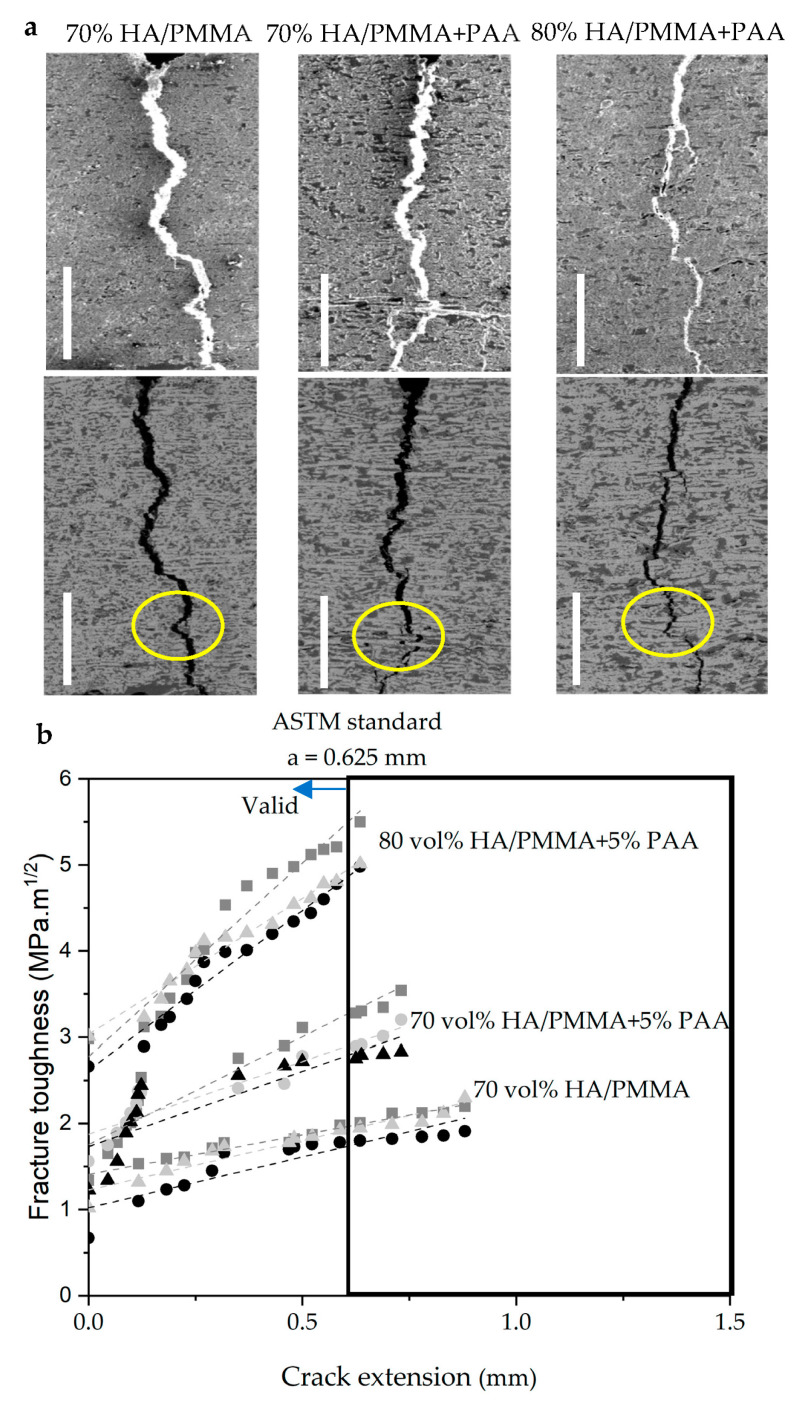
(**a**) The crack-propagation behavior of various samples with different ceramic fractions and polymer systems, observed in situ in a scanning electron microscope (SEM). The yellow circles indicated the crack deflections and ceramic pull-out. Scale bars are 500 µm. (**b**) R–curve of fracture toughness (K_J_) of composites, addition of 5 wt.% PAA to PMMA increased the fracture toughness to 3.023 ± 0.98 MPa·m^1/2^ at 70 vol.% ceramic fraction and increases to the value of 5.27 ± 1.033 MPa·m^1/2^ at higher ceramic fraction of 80 vol.%.

Figure 6a shows the crack-propagation behavior of different composites with different ceramic fractions and polymer systems, observed in situ in a SEM; this allowed real-time observation of the crack propagation and its interaction with the microstructure during toughness measurement. Cracks are deflected at the ceramic/polymer. Stretching and tearing in the polymeric (mortar) can serve as a ligament bridge spanning the crack to carry the load for crack propagation, resulting in additional crack bridging and subsequent pull-out in ceramic (bricks). According to Section 3.1, ceramic bridges can improve the strength and fracture toughness of nacre-like composites by transferring and redistributing stresses to control sliding of individual ceramic layers and prevent delamination. Ceramic bridges increase the roughness of the ceramics, which leads to sliding interference between the ceramic walls during crack propagation, which can improve fracture toughness by energy dissipation of the interference [23,44]. In addition, the polymer phase in a nacre-like composite plays the role of a lubricant phase through which the sliding of the ceramic walls is controlled, leading to high stress release and energy dissipation (Figure 6 indicated with yellow circles). Therefore, as discussed in Section 3.2, silanization can provide stronger interfaces between the ceramic and the polymer and improve the effectiveness of polymer as a viscoelastic adhesive. All of these intrinsic toughening mechanisms are similar to those operating in natural nacre and cortical bone [8,45]. 

Figure 6b shows the K_J_ curve based on crack extension of three composites groups with different ceramic fractions and different polymer phases. The fracture toughness of the composites was measured by determining the area under the load–displacement curve and dividing by the area of the fracture surface. All composites show the resulting R-curve behavior, with the average value of fracture toughness increasing to 3.023 ± 0.98 MPa·m^1/2^ by adding 5 wt.% PAA at 70 vol.% ceramic fraction compared to the composite with the same ceramic fraction containing PMMA. By increasing the ceramic fraction to 80 vol.% in the composite of PMMA + 5 wt.% PAA, the fracture toughness increases to 5.27 ± 1.033 MPa·m^1/2^, which is quite close to toughness behavior of the cortical bone. 

## 4. Discussion

The main goal of this research is to study and fabricate newly designed bone implants that are bioactive and biomimetic in addition to having high strength and fracture toughness. These new implants can improve the efficiency of bone implants and save patients from further problems and surgeries. Taking a cue from nature, a nacre-like composite in which brittle materials such as ceramics can form a tough and strong composite by designing the microstructure. As a result, a hydroxyapatite (HA)/polymer composite with brick-and-mortar structure was developed based on bi-directional freeze-casting method. These HA/polymer composites are bioactive and, because they mimic the microstructure and mechanical properties of cortical bone, can be considered a good candidate for a new bone implant. 

The effective parameters in composite materials can be divided into three main groups, ceramic, interfacial, and polymer. The higher the percentage of ceramic fractions, the thicker and denser the ceramic wall, resulting in higher density of mineral bridges, and higher flexural and compressive strength. The microstructural characterization of composites with different ceramic fractions shows that by increasing the ceramic fractions from 60 vol.% to 80 vol.%, the ceramic walls become thicker and longer, and the density of ceramic bridges increases from 5.89 ± 1.56 to 23.03 ± 2.07 (%) with increasing the ceramic fractions from 60 vol.% to 80 vol.% (see yellow arrows in Figure 1d). The strain-stress curve shows that all the composites without considering the ceramic components exhibit ductile failure caused by the brick-and-mortar microstructure of the composites. The flexural strength and Young’s modulus increase from 115 ± 2.67 MPa and 14.36 ± 2.38 GPa to 130 ± 5.82 MPa and 19.75 ± 2.38 GPa, for ceramic fraction of 70 vol.% and 80 vol.%, respectively. These results are consistent with other nacre-like composites. Sana et al. reported that the flexural strength of Alumina/polymer nacre-like composite increases from 145.77 to 172.65 MPa when the ceramic fraction is increased from 70 vol.% to 76 vol.% [29]. Tan et al. show that increasing the ceramic fractions of zirconia in a nacre-like 3Y–TZP/polymer composite increases the density of mineral bridges and Young’s modulus [6]. 

In addition to the ceramic phase, the interface is also an effective parameter for the development of a tough and strong nacre-like composite. In order to obtain a stronger interface between ceramic and polymer, ceramic surfaces must be grafted to establish the covalent bonds with polymer. The silane-grafted surfaces of HA grafted in 50 wt.% ɣ–MPS for 24 h, as shown in Figure 2a,e, exhibit stretch bonds of carbon–carbon double bonds (C=C), carbon–oxygen double bonds (C=O), and carbon–hydrogen stretch bonds (C–H) with hydrophobic surfaces. This results in covalent bonds of the ceramic with the monomer infiltrated into the scaffolds, giving the flexural strength of 117 ± 1.89 MPa shown in Figure 3, which is higher than that of the composite non-grafted and the composite grafted at the concentration of 25 wt.% for 24 h. Thus, the flexural strength of the composite materials correlates with the interface, and the interface can be improved by grafting or silanization of ceramic surfaces at a suitable level. Olga et al. reported that silanization affects the mechanical properties of the composite HA/PMMA. The hydroxyapatite particles were treated with a silane coupling agent, so that the adhesion between HA and PMMA was improved and the compressive strength increased [46]. Launey et al. show that grafted alumina/PMMA has higher mechanical properties than non-grafted. For a composite with a constant ceramic wall thickness of 20 µm, the flexural strength was 90 MPa in non-grafted composite and 112 MPa for grafted composite [23].

The polymer composition affects the mechanical properties of the final composite. As can be seen in Figure 4a, mixing PAA with PMMA increases the flexural strength of polymer compared to PMMA; however, this improvement has an optimum value of 5 wt.%; beyond that, there is a downward trend. The FTIR analysis shown in Figure 4b shows that mixing PAA with PMMA forms hydroxyl groups on the surface of the polymer phase, which can also be introduce new bonds in interface. As shown in Figure 4b, O-H group on a surface of PAA can interact with free or residue groups on a surface of ceramic, which enhances the strength of interface, as well. The X-ray diffraction (XRD) spectra reveal that the crystalline phase remains unchanged subsequent to both silanization and polymerization processes, as illustrated in Figure 4c. Figure 5 shows how the mechanical properties of the composites change when PAA is mixed with PMMA. The flexural strength increases from 130 ± 5.82 to 158 ± 7.02 MPa, the compressive strength improves from 167.5 ± 2.87 to 189.09 ± 6.45 MPa, and the Young’s modulus increases from 19.75 ± 2.38 GPa to 24 ± 4.34 GPa. All this is due to the improvement in the flexural strength of the neat polymer, as shown in Figure 4a, and improvement in the interface due to the introduction of hydroxyl groups into the interface. The results of other work show that the addition of PAA to PMMA increases the elongation of the polymer and improves the mechanical properties as a copolymer. In addition, the number of polymer chain anchors was increased, and the interface interaction becomes stronger. Therefore, PAA molecules can act as bridges between polymer and ceramic, resulting in higher interfacial bonding force, and significant improvement in mechanical strength. In the absence of PAA, the interfacial strength is relatively weak, resulting in relatively low flexural strength [26].

Fracture toughness is an important feature for mimicking bone implant development because osteons play a role of toughening microelement in cortical bone. The toughness of cortical bone is the result of competition between intrinsic and extrinsic mechanisms operating in this tissue [2]. A nacre-like composite as a result of brick-and-mortar microstructure shows an intrinsic toughening mechanism like cortical bone. Hydroxyapatite itself, like other ceramics, is a brittle material that exhibits low toughness and has catastrophic failure. The fabricating nacre-like HA/polymer composites resulted in extrinsic toughness mechanisms in the composite, where the brick-and-mortar microstructure reduces the crack-driving force behind the crack tip. In addition, the polymer phase can contribute to plastic deformation and act as a lubricant.

Figure 6a shows the in situ images SEM for composites with ceramic fraction of 70 vol.% infiltrated with PMMA and PMMA + 5 wt.% PAA; also, the composites with higher ceramic fractions of 80 vol.% in the same polymer compositions of PMMA + 5 wt.% PAA, all composites show tortuous cracking. In addition, crack deformation, pull-out of ceramic bricks, and frictional sliding between ceramic walls were also observed, all of which are extrinsic toughening mechanisms. Fracture toughness characterization of HA/polymer composites showed a rising R–curve behavior in different ceramic fractions of 70 vol.%, and 80 vol.% and polymer composition approved the hypothesis of a rising R–curve behavior of nacre-like composites as shown in Figure 6b. As shown, the addition of PAA to PMMA increases the fracture toughness of the composite from 1.99 ± 0.78 to 3.023 ± 0.98 MPa·m^1/2^ which is caused by higher strength in the composites consist of copolymer of PMMA + PAA. In turn, increasing the ceramic fraction from 70 vol.% to 80 vol.% in the same polymer compositions, the fracture toughness reaches 5.27 ± 1.033 MPa·m^1/2^. Hao et al. showed the same results for HA/PMMA nacre-like composite, where the fracture toughness improved with increasing ceramic fraction. The value of fracture toughness increases with the increase in ceramic fractions [5]. Therefore, it can be concluded that the fracture toughness correlates with the ceramic fraction. It could be explained by the extrinsic toughness mechanisms mentioned above that when the ceramic fraction is increased from 60 vol.% to 80 vol.%, the density of the ceramic bridges and wall thickness increase, resulting in thinner polymer layers and increasing the stress-relieving lubricating effect. Hongbo et al. show the same results by decreasing the thickness of the polymer layers and thus increasing the fracture toughness in a nacre-like composite material [34].

## 5. Conclusions

In summary, a good combination of high strength and toughness was achieved in a nacre-like, bioactive HA-polymer composite fabricated by bi-directional freeze-casting. Ceramic, interface, and polymer are the main effective parameters leading to potential composites for future bone implants. Increasing the ceramic fractions in the brick-and-mortar microstructure increases the density of the ceramic bridges, resulting in better mechanical properties. The composites HA/PMMA with ceramic fractions of 60 vol.%, 70 vol.%, and 80 vol.% exhibit flexural strength of 52.68 ± 3.78, 115 ± 2.67, and 130 ± 5.82 MPa, respectively. The nacre-like HA/polymer composite exhibits a-rising R–curve behavior caused by the brick-and-mortar microstructure. The addition of 5 wt.% PAA to PMMA increases the number of polymer chains and the chains are more elongated, resulting in higher mechanical properties of the composites compared to pure PMMA. The flexural strength and fracture toughness were improved by about 20% to 158 ± 7.02 MPa and 5.27 ± 1.033 MPa·m^1/2^, respectively, at 80 vol.% ceramic fraction. The strong, tough, bone-matching and bioactive materials are expected to out-perform the materials currently used in orthopedics such as spinal fusion and bone fracture fixation implants.

## Data Availability

Data are available upon request from the corresponding author.
